# Graph Empirical Mode Decomposition-Based Data Augmentation Applied to Gifted Children MRI Analysis

**DOI:** 10.3389/fnins.2022.866735

**Published:** 2022-07-01

**Authors:** Xuning Chen, Binghua Li, Hao Jia, Fan Feng, Feng Duan, Zhe Sun, Cesar F. Caiafa, Jordi Solé-Casals

**Affiliations:** ^1^Department of Artificial Intelligence, Nankai University, Tianjin, China; ^2^Computational Engineering Applications Unit, Head Office for Information Systems and Cybersecurity, RIKEN, Saitama, Japan; ^3^Instituto Argentino de Radioastronomía, Consejo Nacional de Investigaciones Científicas y Técnicas – Centro Científico Tecnológico La Plata/Comisión de Investigaciones Científicas – Provincia de Buenos Aires/Universidad Nacional de La Plata, Villa Elisa, Argentina; ^4^Department of Psychiatry, University of Cambridge, Cambridge, United Kingdom; ^5^Data and Signal Processing Research Group, University of Vic-Central University of Catalonia, Vic, Spain

**Keywords:** GEMD, MRI, gifted children, structural connectivity, BrainNetCNN

## Abstract

Gifted children and normal controls can be distinguished by analyzing the structural connectivity (SC) extracted from MRI data. Previous studies have improved classification accuracy by extracting several features of the brain regions. However, the limited size of the database may lead to degradation when training deep neural networks as classification models. To this end, we propose to use a data augmentation method by adding artificial samples generated using graph empirical mode decomposition (GEMD). We decompose the training samples by GEMD to obtain the intrinsic mode functions (IMFs). Then, the IMFs are randomly recombined to generate the new artificial samples. After that, we use the original training samples and the new artificial samples to enlarge the training set. To evaluate the proposed method, we use a deep neural network architecture called BrainNetCNN to classify the SCs of MRI data with and without data augmentation. The results show that the data augmentation with GEMD can improve the average classification performance from 55.7 to 78%, while we get a state-of-the-art classification accuracy of 93.3% by using GEMD in some cases. Our results demonstrate that the proposed GEMD augmentation method can effectively increase the limited number of samples in the gifted children dataset, improving the classification accuracy. We also found that the classification accuracy is improved when specific features extracted from brain regions are used, achieving 93.1% for some feature selection methods.

## Introduction

Intelligence can be seen as the ability to recognize and understand reality and use knowledge and experience to solve problems such as memory, observation, imagination, thinking, and judgment. Gifted children are regarded to have higher intelligence and perform better in attention, language, mathematics, verbal working memory, shifting, and social problem-solving (Bucaille et al., 2022). At the same time, gifted children demonstrate high working memory capacity and more effective executive attention (Aubry et al., 2021). They also have significant differences in cognitive flexibility function and problem-solving and reasoning (Rocha et al., 2020).

Gifted children have higher intelligence and learn faster than others, probably due to differences in neurophysiology (Gross, 2006). Neurological differences mean that gifted children may experience neurodevelopmental trajectories different from normal children, leading to a greater connection of neuronal pathways (Navas-Sánchez et al., 2014). Gifted children have larger subcortical structures and more robust white matter microstructural organization between those structures in regions associated with explicit memory (Kuhn et al., 2021). They are also characterized by highly developed functional interactions between the right hemisphere and excellent cognitive control of the prefrontal cortex, enhanced frontoparietal cortex, and posterior parietal cortex (Wei et al., 2020). Ma et al. found that gifted children have network topological properties of high global efficiency and high clustering with a low wiring cost and a higher level of local connection density (Ma et al., 2017). Gifted children's structural brain network has a more integrated and versatile topology than normal children (Solé-Casals et al., 2019).

Based on previous work on the brain neuroscience of gifted children, we believe that it is significant to identify gifted children through the structure of their brains. In the past decades, many neuroscientists have tried to understand the brain mechanisms and proposed many types of neuroimaging techniques, such as magnetic resonance imaging (MRI), functional magnetic resonance imaging (fMRI), and diffusion tensor imaging. In recent years, deep learning algorithms have achieved good results in processing these types of signals. Abdelaziz Ismael et al. proposed an enhanced deep learning approach, residual networks, for brain cancer MRI images classification and achieved 99% accuracy (Abdelaziz Ismael et al., 2020). Sarraf et al. used convolutional neural network (CNN) architectures Lenet-5 and GoogleNet to classify fMRI data of Alzheimer's disease subjects and normal controls, and the accuracy of the test dataset reached 96.85% (Sarraf and Tofighi, 2016). The BrainNetCNN is proposed to predict clinical neurodevelopmental outcomes by brain networks (Kawahara et al., 2017). It utilizes structural brain networks' topological locality to create edge-to-edge (E2E), edge-to-node (E2N), and node-to-graph (N2G) convolutional filters, which makes it perform well on human brain data classification. Leonardsen et al. proposed that neural network is able to identify subject brain from its MRI (Leonardsen et al., 2022).

The deep learning technology is notable for its impressive performance and generalization capability, but the number of effective samples in the medical imaging dataset is usually small, leading to performance degradation. The training model needs large amount of data to avoid overfitting (Caiafa et al., 2020). However, obtaining enough MRI data is not easy. The acquisition and preprocessing of brain data are more difficult than image and voice data, for example. It is difficult to find gifted children in our daily life. The number of gifted children is small, especially those whose IQ test score is higher than 140. In this work, we use a sample of 29 children, from which the MRI was obtained. The brain was parcellated into 308 regions and from each region 7 morphometric features were extracted. Hence, we have a total of 2,156 features per subject (7 morphological features by 308 brain regions). Training a model in such a small and high-dimensional MRI dataset is complicated. Therefore, we focus on MRI data augmentation to improve model training. Data augmentation has proven to be useful in MRI, improving the accuracy of schizophrenia classification by 5% (7–8% relative improvement using augmentation) (Ulloa et al., 2015). Also, Nguyen et al. proposed a data augmentation method synthesizing a new fMRI image by performing a T1-based coregistration to another subject's brain in native space. This method was tested on antidepressant treatment response fMRI and demonstrated a 26% improvement in predicting response using augmented images (Nguyen et al., 2020). Previous work proves that increasing the amount of neuroimaging data through an appropriate data augmentation method can significantly improve the accuracy of deep learning classification.

In our MRI dataset, we propose to use a data decomposition method, graph empirical mode decomposition (GEMD) (Tremblay et al., 2014). GEMD is an adaptation to graph signals of the well-known empirical modal decomposition (EMD) (Huang et al., 1998). EMD has some variants, such as GEMD, masking EMD, ensemble-EMD (EEMD), and multivariate EMD (MEMD). Masking EMD, EEMD, and MEMD can primarily alleviate the mode mixing problem, and masking EMD and MEMD can perform spatiotemporal reconstruction of active sources (Muñoz-Gutiérrez et al., 2018). GEMD improves many aspects of the critical points of EMD, namely, extrema, interpolation, and stopping criterion (Tremblay et al., 2014). Because a parcellation of 308 brain regions is used, which can help to build a brain region connection graph, GEMD is the best choice for our work, as we will base our data augmentation on the decomposition-recombination strategy first presented in Dinarès-Ferran et al. (2018) for EEG signals. To our knowledge, this is the first time this technique has been used on MRI data. To compare the results of the proposed method, we also generate artificial samples through a more classical approach, the synthetic minority over-sampling technique (SMOTE) (Chawla et al., 2002).

In this work, the BrainNetCNN is used as a deep learning classifier. The main motivation for using a deep learning method is that the MRI data can then be fed directly into the model without the need for any feature selection method. This is an important aspect to keep in mind as feature selection methods are usually very database-dependent, and the results could drop if the database is changed. We train the BrainNetCNN for the classification task, showing that a well-trained classification model can increase the classification accuracy from 55.7 to 78% when using artificial data. Moreover, in Zhang et al. (2021), a hybrid selection method of morphological features and brain regions on the same gifted children dataset was derived. They used a completion method, simultaneous tensor decomposition, and completion (STDC), for outlier correction. After tensor completion, several feature selection methods were applied to the training set to explore which morphometric features and brain regions could perform better in the classification step. Based on their methodology, we used GEMD to generate artificial data on Zhang et al.'s work to achieve an accuracy of 93.1% on the F-score (FS), combined feature selection, and rank FS method.

The rest of the article is organized as follows. the materials and methods and the details of the experiments are introduced. Then, the experimental results are discussed, followed bydiscussion. Finally, the conclusions are summarized.

## Materials and Methods

**Figure 1:**
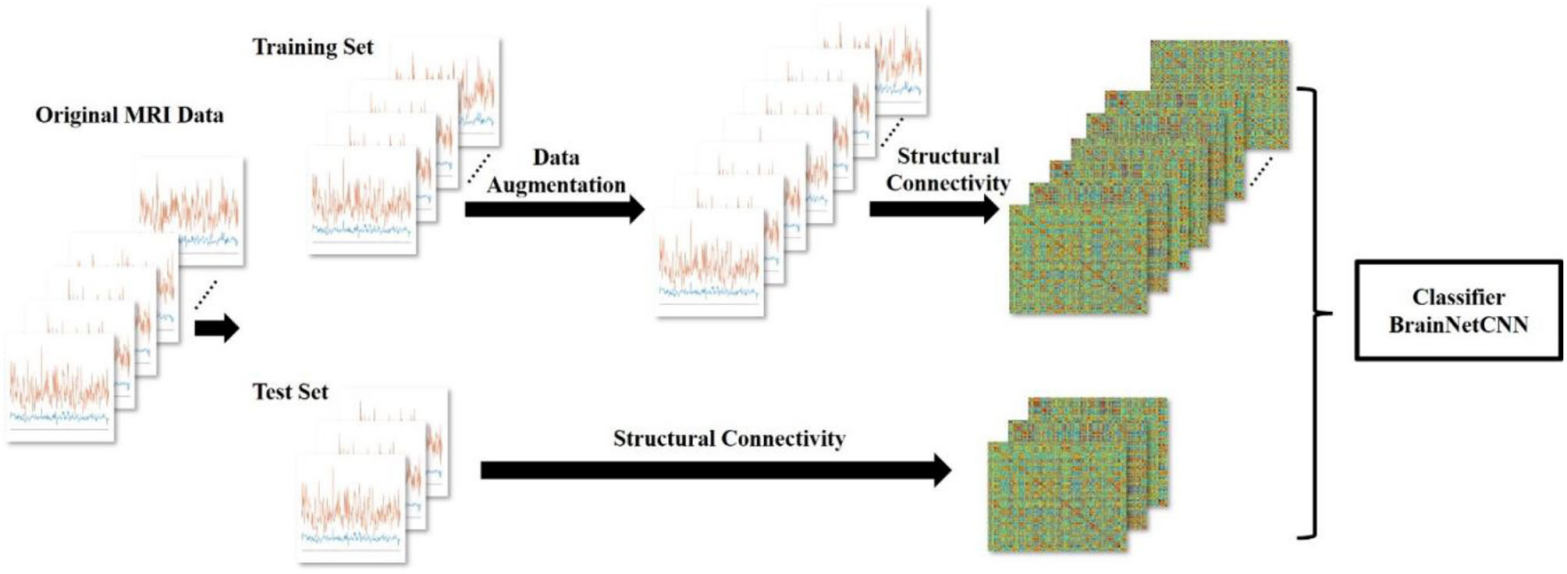
Overview of the process for the data augmentation of the gifted children MRI dataset using GEMD. From left to right, the seven curves representing the morphometric features in the 308 regions of the brain, for each subject (original MRI data). Then, the data are split into training and test sets; the training set is augmented using GEMD; the structural connectivity of the data is calculated and used to feed the deep learning model. Finally, the structural connectivity is also derived for the samples of the test set to demonstrate the capability of the classifier.

The overall experimental process is shown in Figure 1. In this section, we introduce the six parts in order. The details of the data are first described. Then, we show the brain region atlas and the morphometric features. After that, the basic algorithm principle of GEMD will be provided. Then, the data augmentation with GEMD is introduced. The following is the structural connectivity (SC) analysis, which converts MRI images into a correlation matrix. Finally, we introduce a deep learning network, the BrainNetCNN, as a classifier.

### Gifted Children MRI Dataset

The MRI dataset of gifted children contains 29 healthy, right-handed male subjects without neurological diseases (Solé-Casals et al., 2019). We refer to this dataset as the UVic-gifted children dataset (UVic-GC dataset). There is no significant age difference between the two groups. Gifted children have a high IQ and outstanding performance in various tasks such as spatial, numerical, reasoning, verbal, and memory (Gras et al., 2010). The criteria for gifted group included having an IQ in the very superior range (≥140). Gifted children also had a performance above the 90th percentile in three of the following aptitudes, namely, spatial, numerical, abstract reasoning, verbal reasoning, and memory. More details on the dataset can be found in Solé-Casals et al. (2019). Table 1 summarizes the details of the dataset. Using similar procedure and scanning parameters, all participants underwent examinations in a 3 T MRI scanner (Magnetom Trio Tim, Siemens Medical Systems, Germany). The raw (anonymized) MRI data are available in the OpenNeuro repository (https://openneuro.org/datasets/ds001988).

**Table 1:** Membership information of gifted children MRI dataset.

**Group**	**Gifted group**	**Control group**
Average IQ	148.80 ± 2.93	122.71 ± 3.89
Average age	12.03 ± 0.54	12.53 ± 0.77
Sample size	15	14

### Brain Region Atlas and Morphometric Features

In our study, the brain is divided into 308 cortical regions following previous work (Romero-Garcia et al., 2012). The parcellation atlas is based on the Desikan-Killiany Atlas (68 cortical areas). Each area defined in the Desikan-Killiany atlas is subdivided into spatially contiguous areas through a backtracking algorithm available in FreeSurfer (Desikan et al., 2006). The size of each area is approximately equal to 500 mm^2^.

**Figure 2:**
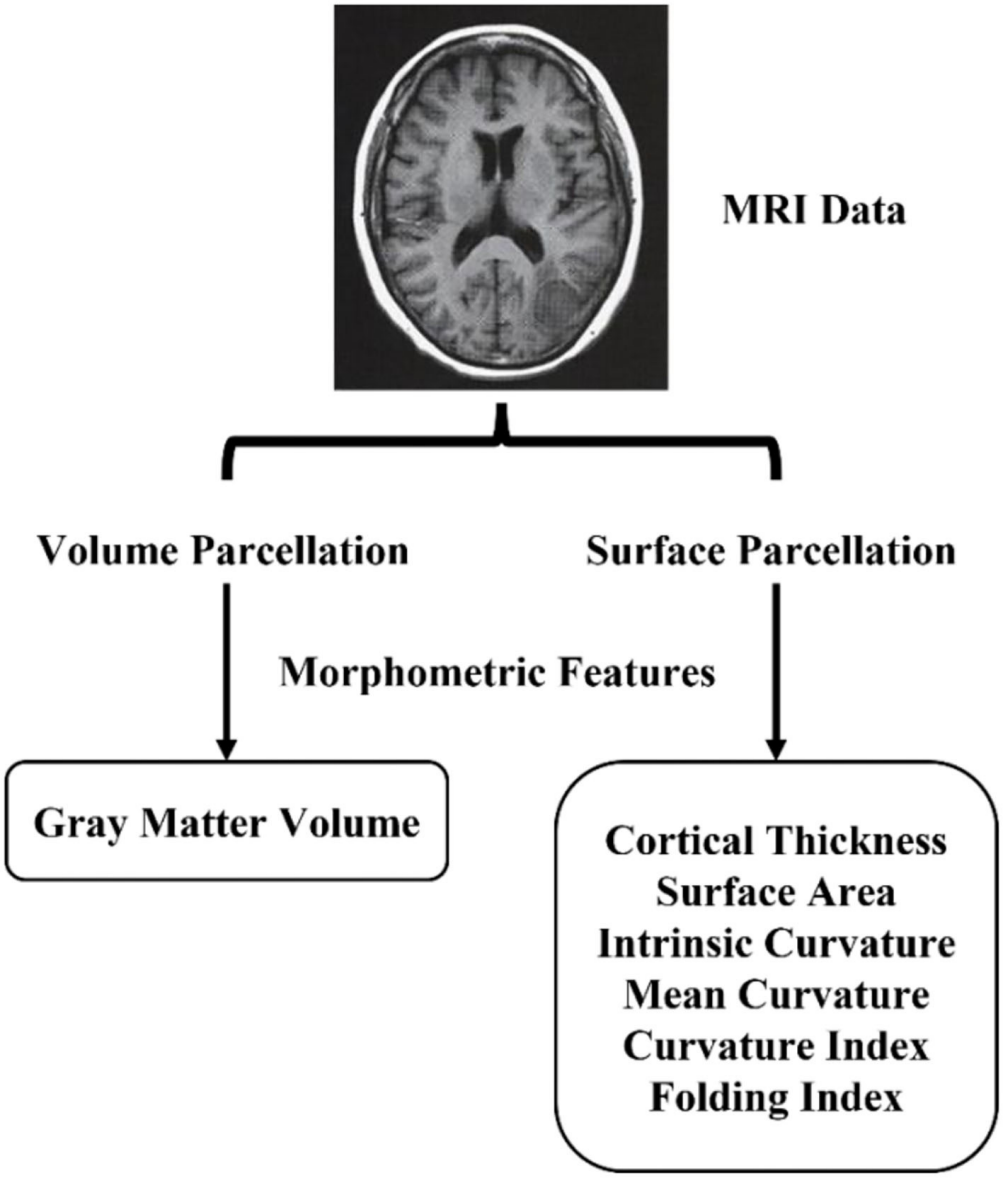
Morphometric features extraction pipeline.

The original feature matrix includes seven morphological features measured in each of the 308 brain regions. Figure 2 shows the morphological features such as gray matter volume, cortical thickness, surface area, intrinsic curvature, mean curvature, curvature index, and fold index.

### Graph Empirical Mode Decomposition

Empirical modal decomposition can decompose a signal into a set of intrinsic mode functions (IMFs), each covering different frequency bands by interpolating the extremes in the time series (Huang et al., 1998). The IMFs have two characteristics, namely, (1) the number of its zero crossings must be equal or differ up to one compared to its number of extrema and (2) IMFs' upper and lower envelopes must be symmetric to zero. When all the IMFs of the original signal are extracted, the iterative process is terminated. GEMD is an adaptation of the classical EMD for graph signals (Tremblay et al., 2014). It improves many aspects of the critical points of EMD, namely, extrema, interpolation, and stopping criterion.

For the graph creation, the set of *N* regions is used as nodes for the graph. A weighted graph parameter δ is used to define edges in the graph. Only pairs of regions (*i, j*) at a distance *d*_*i, j*_, shorter than δ, are connected by an edge, with weight $w_{i,j}\;=\;\exp\left(-{d^{2}}_{i,j}/2\delta^{2}\right)$. The distance *d*_*i, j*_ is the Euclidean distance in the features space. In that case, we get a graph *G* = (*N, E*), where *E* is the set of edges. The adjacency matrix *A*, which contains all the weights *w*_*i, j*_ connecting the nodes, is also needed. We use the 3D coordinate points of 308 brain regions to calculate the adjacency matrix for the 308 brain regions graph.

For the definition of local extrema, a node *n* will be a local minimum (or maximum) if for all its neighbors in *G*, *x*(*n*) < *x*(*m*) [or *x*(*n*)>*x*(*m*), where *x*(*n*) and *x*(*m*) represent the value of one of the features in the *n*_*th*_ and *m*_*th*_ brain regions]. Once the extremes have been obtained, the graph signal is interpolated to get the lower and upper graph envelopes needed to derive the IMFs.

To maintain the hypothesis-free nature of the classical EMD method, interpolation is regarded as a discrete partial differential equation on the graph (Grady and Schwartz, 2003). As the envelope is a slowly changing component, the interpolation signal *s* needs to minimize the total graph change, *s*′*Ls*, where *L* is the graph Laplacian matrix under the constraint that the graph signal value of the known vertex remains unchanged. Let *K* denote the set of vertices of the known graph signal, and *U* denote the set of unknown vertices. Then, to calculate the new, interpolated, graphical signals, we need to solve the following equation minimize *s*′*Ls* subject to:


(1) $$\begin{array}{r} s\left(K\right)=x\left(K\right) \end{array}$$

Through simple rearrangement of vertices, *s* can be rewritten as $s^{{}^{\prime }}=\left[{s^{{}^{\prime }}}_{K}\;{s^{\prime }}_{U}\right]$ in its equivalent vector expression, where *s*_*K*_ and *s*_*U*_ are the vector representations of *s*(*K*) and *s*(*U*), and the rearranged Laplacian matrix $L=\left[\begin{array}{cc} L_{K} & R\\ R^{\prime } & L_{U} \end{array}\right]$. Finally, the graph interpolation is a Dirichlet problem on the graph, and its solution depends on the following linear equation (Kalaganis et al., 2020):


(2) $$\begin{array}{r} L_{U}S_{U}=-Rs_{k} \end{array}$$

We refer the reader to Grady and Schwartz (2003) and Kalaganis et al. (2020) for a detailed explanation of the graph interpolation method. With the mentioned elements, the sifting process can be modified easily. We set the parameter of the stopping criterion, which was defined in Tremblay's work (Tremblay et al., 2014), as follows: stop the loop (steps 4–8 in the following algorithm) as soon as the energy of the average envelope *z* (computed in the step 6) is lower than the energy of the analyzed signal *x*_*i*_ divided by 1,000.

**Figure 3:**
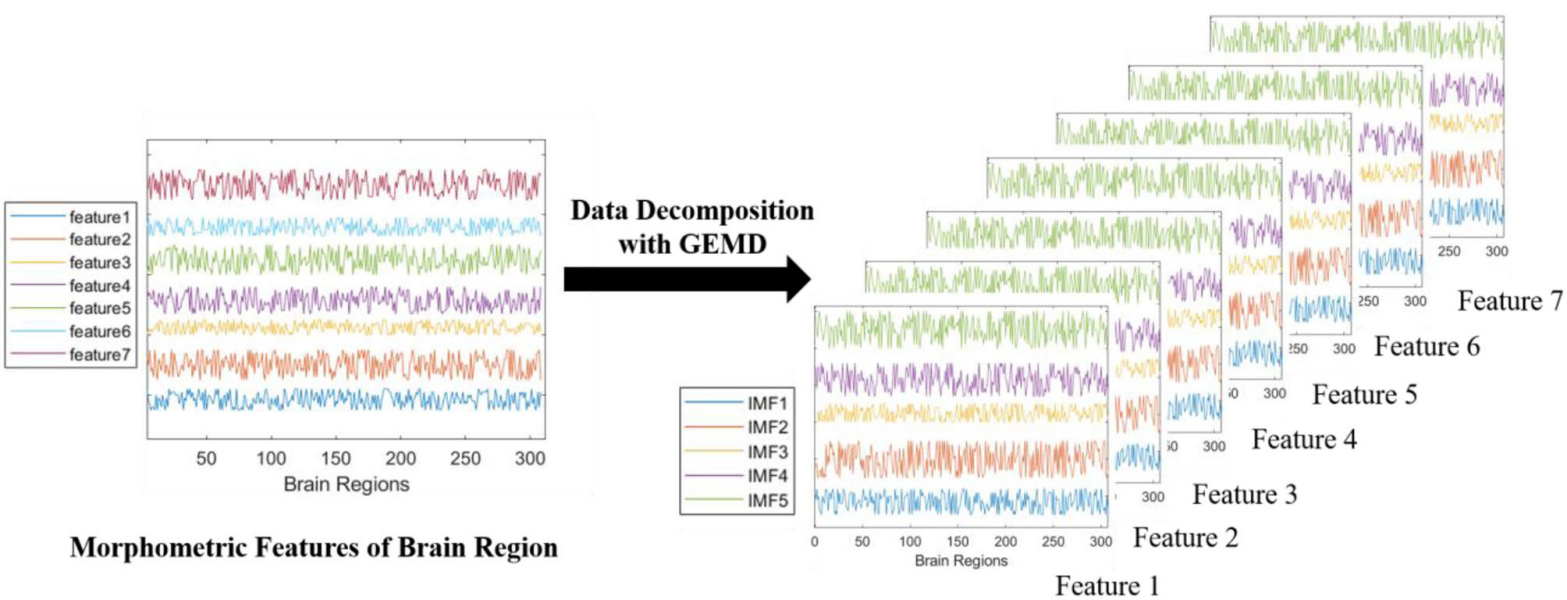
The progress of data decomposition with GEMD. GEMD can decompose feature series (different color lines on the left) into IMFs simultaneously. Here, we use seven morphometric features from MRI data as a decomposition example. Every feature is decomposed into four or five IMFs (different color lines on the right). If only four IMFs are decomposed from raw data, zero-padding will be used to have a total of five IMFs in all the decompositions, so that the data augmentation can proceed successfully.

After defining the graph extremum and interpolation process, the classic EMD algorithm can easily be extended to graph signals. The process of data decomposition with GEMD is shown in Figure 3. The GEMD algorithm (Tremblay et al., 2014) is defined as follows:

- Step 1: Create the adjacency matrix *A* for the graph *G*;
- Step 2: Initialize *m* = *x*_*i*_;
- Step 3: While *m* does not meet the stopping criterion, repeat step 4 to step 8;
- Step 4: Detect the local extreme of *m* ;
- Step 5: Interpolate the upper and lower extremes of *m* and get the envelop*e*_max_and *e*_min_;
- Step 6: Calculate the average envelope $z=\;\frac{e_{\min}+e_{\max}}{2}$;
- Step 7: Subtract the average envelope from the signal: *m* = *m* − *z*;
- Step 8: Set *d*_*i*+1_ = *m* and *x*_*i*+1_ = *x*_*i*_ − *m*;
- Step 9: If *m* meets the stopping criteria: stop the decomposition and terminate, return stored IMFs, and get [Mathtype-mtef1-eqn-3.mtf].

### Data Augmentation

**Figure 4:**
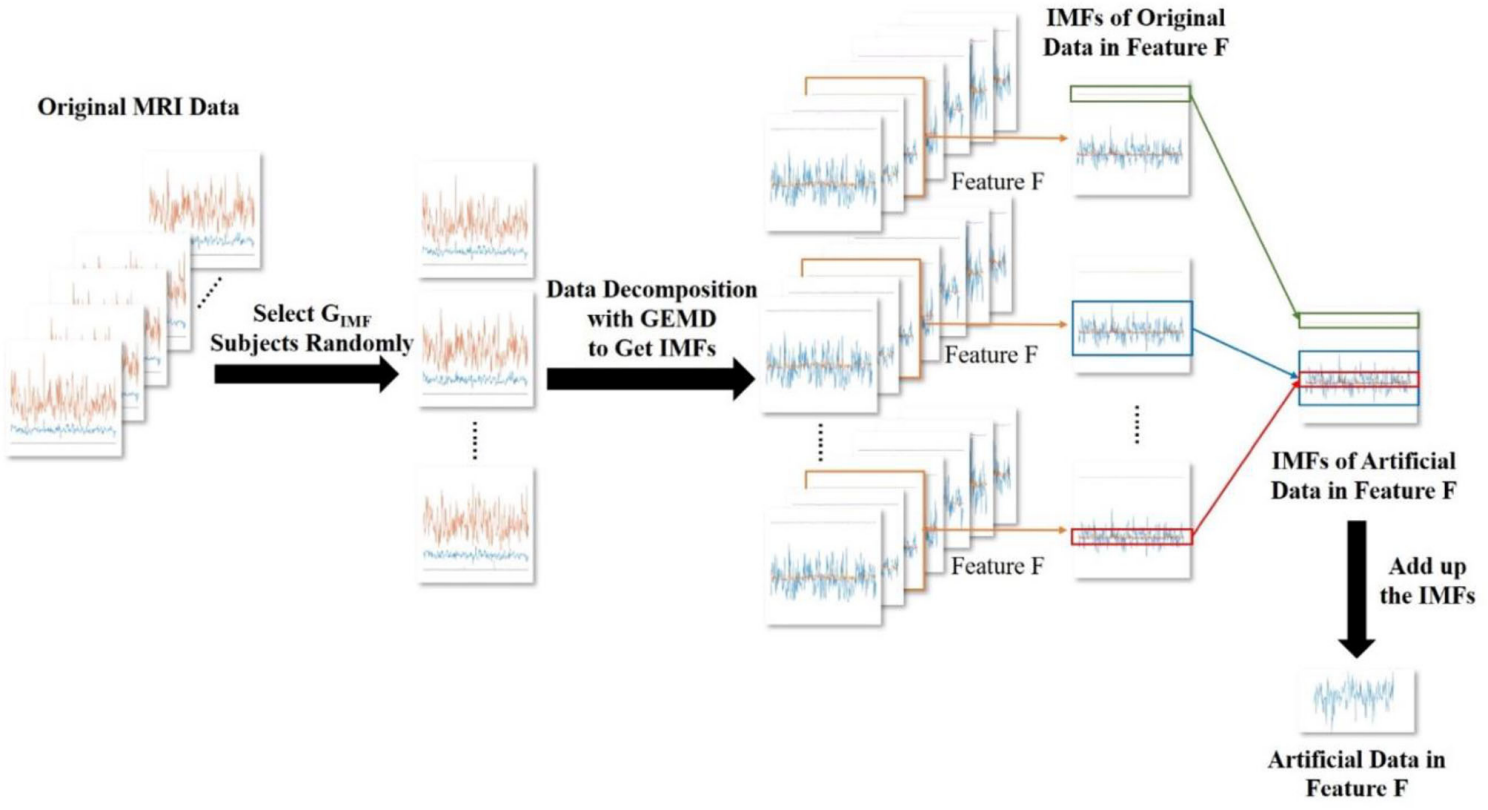
The generation of artificial MRI data. Here, we generate the artificial data in feature F as an example. We randomly select *M* subjects from the original MRI data. Then, we obtain the IMFs, which are decomposed with GEMD. The IMFs decomposed form feature F of the *M* randomly selected subjects are recombined. Then, they are added up to obtain the artificial data of the feature F.

The MRI dataset contains *P* = 29 subjects. Therefore, the training set can be regarded as a three-dimensional tensor *T*∈*R*^*BxFxP*^ (*B*: number of brain regions; *F*: number of features; *P*: number of subjects). If the number of subjects in the training set is too small, the model will tend to be overfitted. To overcome the overfitting problem in the UVic-GC dataset, we propose to increase the training set through GEMD. The data augmentation procedure is based on a decomposition-recombination strategy, originally proposed in Dinarès-Ferran et al. (2018), and first used in a deep learning context in Zhang et al. (2019). The data augmentation process is shown in Figure 4. This method has the following steps:

- *Step 1: Data decomposition*.

- Create the adjacency matrix *A* for the graph *G*. In our work, *A* is obtained by calculating the Euclidean distance among the 308 regions.
- Organize the MRI data of all subjects and get the concatenated tensor *T*∈*R*^*BxFxP*^.
- Decompose *T* with GEMD and get $T_{IMF}\in R^{M\times B\times F\times P}$, where *M* is the total number of IMFs (*M* = 5 in our experiments).

- *Step 2: Artificial data generation*.

- Randomly select *M* subjects from one of the groups (gifted group or control group).
- Take one IMF from each subject so that you end up with one IMF from each category (IMF_1_ to IMF_5_), i.e., each subject contributes with one IMF to create the new artificial data. The artificial data of the *n*_*th*_ feature is the sum of the *M* IMFs.

### Structural Connectivity Analysis

After creating artificial samples, we use the original subjects and the artificial to perform the classification. For that purpose, we calculate the SC between features in all the regions. The SC matrix (one matrix per sample, i.e., original subjects and artificial subjects created *via* GEMD) will be used later as the input data for the deep learning classification system. SC represents the data correlation between two brain regions (Qi et al., 2019). Pearson's correlation or coherence is usually used to compute the correlation. We use Pearson's correlation and *z*-score to obtain the SC in this work. We correlate the seven values (morphometric features) of one region with the seven values (morphometric features) of another region. We perform these correlations for all possible pairs, obtaining a 308 ×308 matrix per subject. Assuming two brain region data *x* and *y*, Pearson's correlation (Kotu and Deshpande, 2019) between *x* and *y* can be expressed as follows:


(3) $$\begin{array}{r} c\left(x,y\right)=\frac{S_{xy}}{\sqrt{S_{xx}S_{yy}}} \end{array}$$

where *S*_*xy*_ is the covariance of *x* and *y*, which is defined as,


(4) $$S_{xy}=\sum_{i=1}^{n}\left(x_{i}-x\right)\left(y_{i}-y\right)$$

*S*_*xx*_ and *S*_*yy*_ can be calculated as the variance of *x* and *y*, respectively. After we get the Pearson's correlation of MRI data, *z*-score is used to standardize it. Finally, a three-dimensional tensor of dimensions 29 × 308 × 308 is obtained.

This procedure was introduced by Seidlitz et al. (2018) to estimate the inter-regional correlation of multiple MRI features in a single subject instead of estimating the inter-regional correlation of a single feature measured in multiple subjects (which is done with the structural covariance analysis). Therefore, we end up with an SC matrix per subject.

### Neural Network Classifier

**Figure 5:**
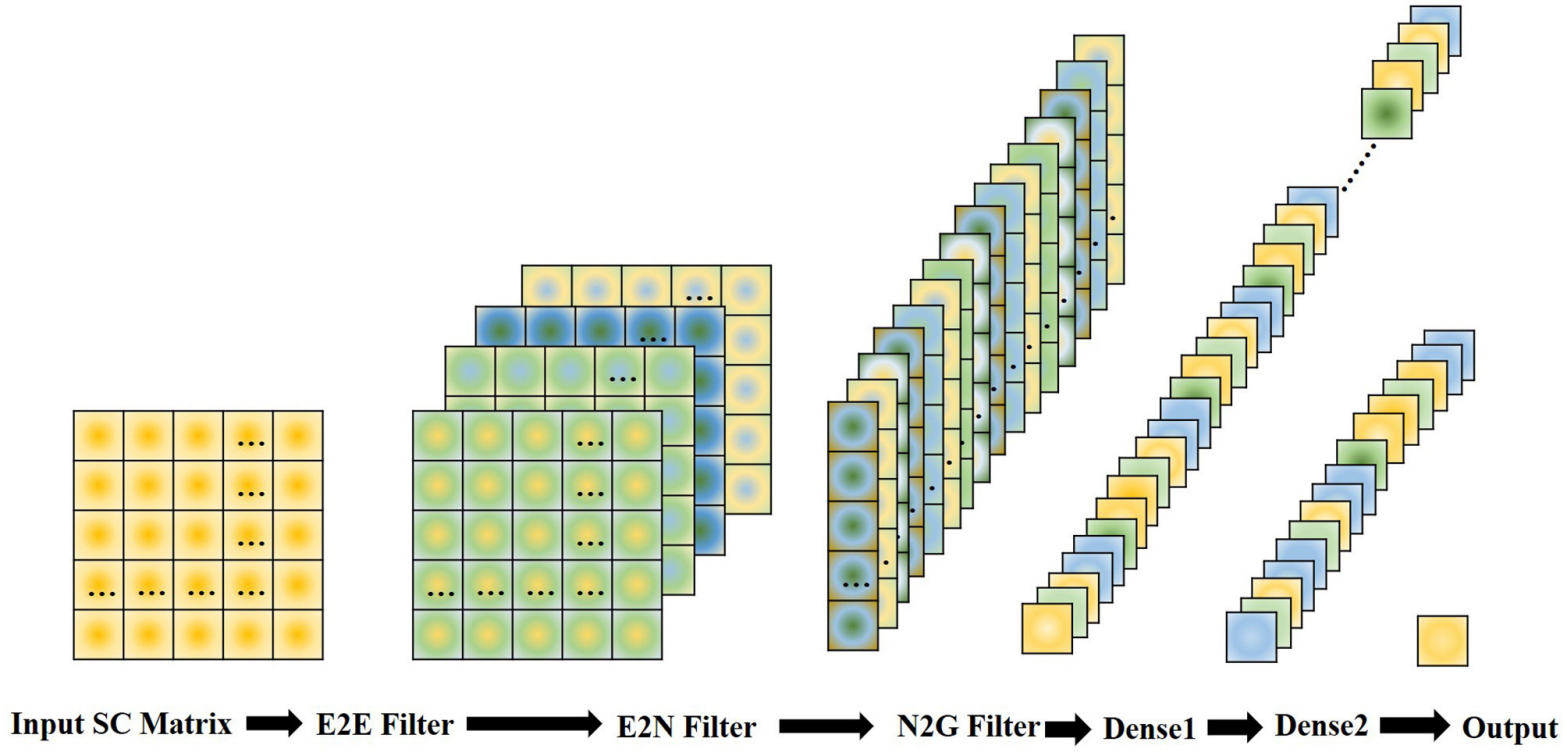
Structure of the BrainNetCNN network.

As the BrainNetCNN (Kawahara et al., 2017) outperforms lots of other methods on structural brain networks datasets, we choose it as a neural network classifier in our experiments. There are three kinds of convolutional filters in BrainNetCNN, namely, E2E, E2N, and N2G filters. They leverage the topological locality of structural brain networks. E2E filter convolves the brain network adjacency matrix and weights edges of adjacent brain regions. E2N filter assigns each brain region a weighted sum of its edges. N2G assigns a single response based on all the weighted nodes. These three filters consist of convolution kernels: kernel $c_{1}\in R^{1\times D}$, $c_{2}\in R^{D\times 1}$. The kernel of the E2E filter is ${c_{E2E}=c}_{1}+c_{2}\in R^{D\times D}$. *D* is the number of nodes in a graph, which corresponds to the number of brain regions in this work. The kernels of the E2N filter and N2G filter are *c*_*E*2*N*_ = *c*_1_, *c*_*N*2*G*_ = *c*_2_. In our experiment, the structure of the BrainNetCNN can be simply expressed as Input (308 × 308 SC matrix) -> E2E (4 channels) -> relu -> E2N (16 channels) -> relu -> N2G (32 channels) -> dense1 (16 channels) -> dense2 (1 channels). This structure is shown in Figure 5. We use the adaptive moment estimation (Adam) optimizer, with learning rate *lr* = 0.001, β_1_ = 0.9, and β_2_ = 0.999. The network is trained using 300 epochs, and the batch size is 32. Considering the size of the dataset, we applied 10-fold cross-validation and repeated the experiment 10 times to get the average accuracy.

## Results

### GEMD Performance on BrainNetCNN

We want to prove that the data augmentation with GEMD can improve the performance of the BrainNetCNN in the classification of the UVic-GC dataset. Therefore, we randomly selected 14 subjects (7 from the gifted group and 7 from the control group) as the original MRI data for the training set. The training set also contains artificial MRI data generated through GEMD from the original data of this training set. The rest of the subjects are used as the test set, containing 15 subjects.

Aiming to study how the number of artificial subjects affects the performance in the training set, we increase the number of artificial samples from 0 to 400 for each group. For each session, the original MRI data are split into the training set and test set. The training set is used to generate the required number of artificial samples. The model is then trained using the original training set and the artificial samples generated from it, and finally the model is tested with the remaining test set. This process is repeated 10 times for each number of artificial samples to get the final average accuracy.

**Figure 6:**
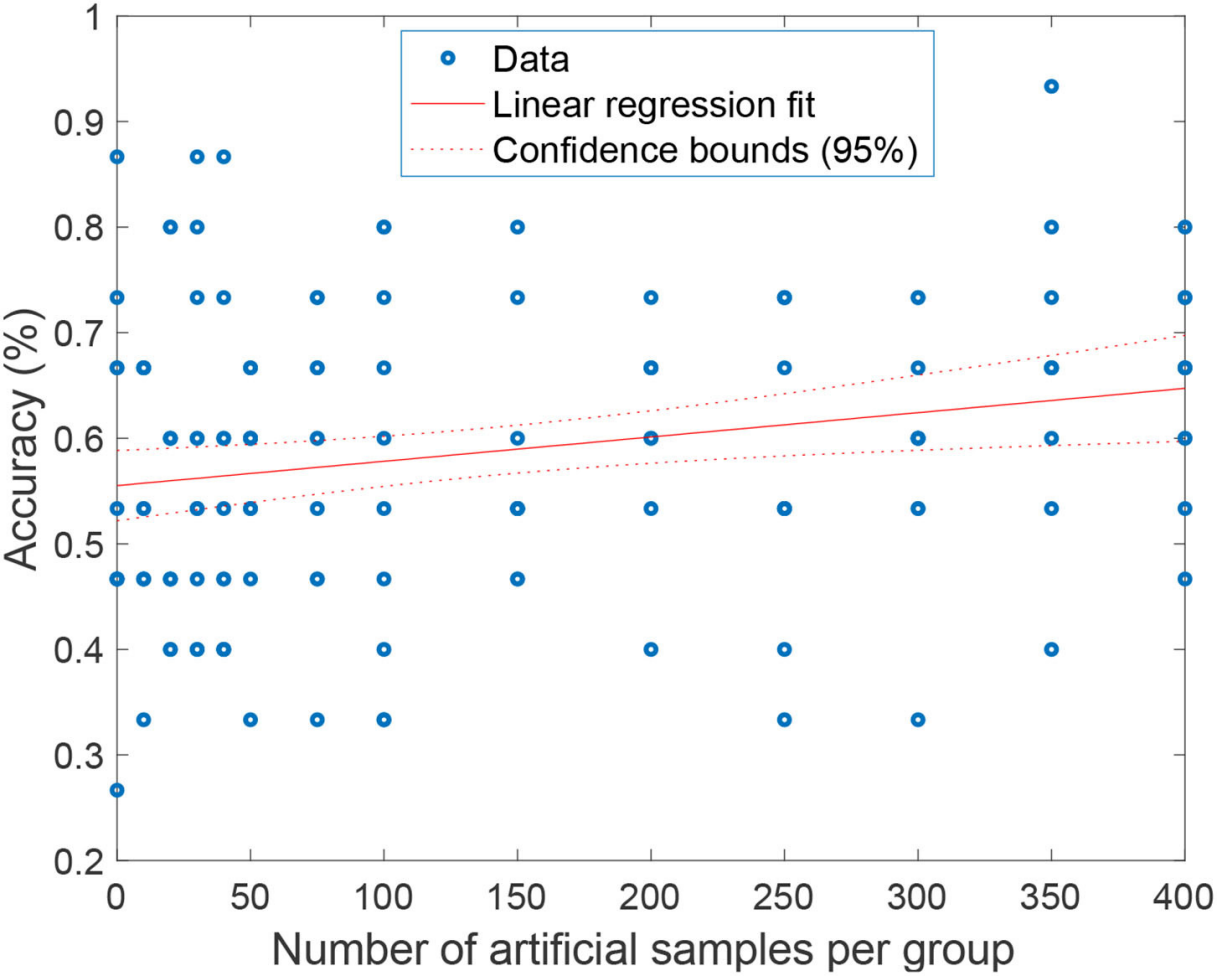
The mean of accuracy and standard deviation with GEMD augmentation for the different number of artificial samples.

In Figure 6, we show the classification accuracy for a different number of artificial samples. As can be seen, the performance of the BrainNetCNN can be improved when adding artificial samples, from 10 artificial samples to 400 artificial samples. The improvement increases when the number of artificial samples increases. Fitting a linear regression model gives us an idea of the expected improvement when adding artificial samples. The model shows a positive trend of gradient *x*_1_ = 0.00023035, with a *p*-value < 0.01. This means that we should expect a 2.3% increase in the accuracy per 100 artificial samples added. The BrainNetCNN has the best mean accuracy performance at 66.7% when the number of artificial samples is 350, while without GEMD, the mean accuracy is only 56%. This is an increase of 10.7%, slightly better than the 8% predicted by the linear model.

**Figure 7:**
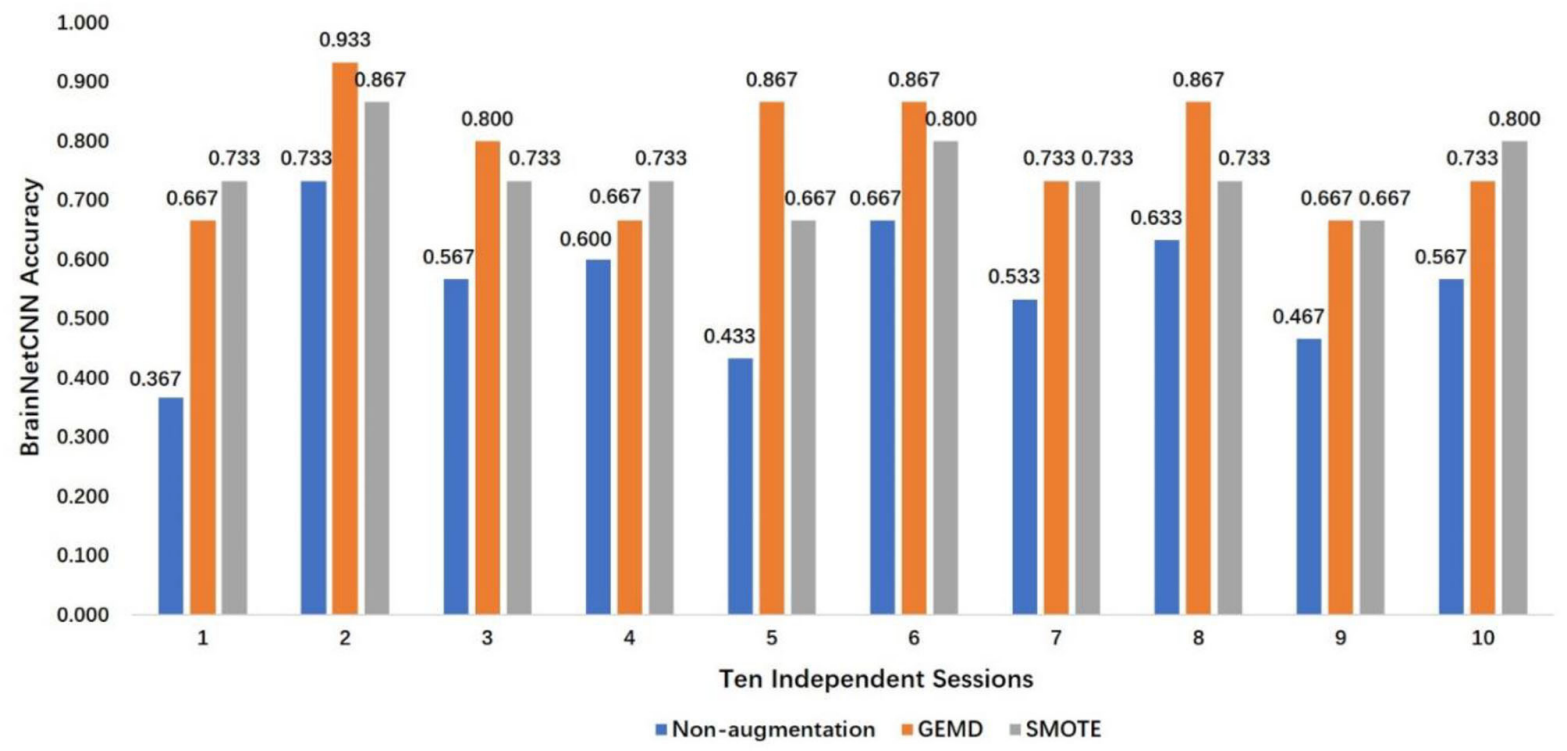
Best accuracy using GEMD, SMOTE, and non-augmented case in 10 independent sessions.

The SMOTE is also used. The results are compared and depicted in Figure 7, which presents the best accuracy with GEMD, SMOTE, and non-augmented cases (baseline) in 10 different sessions. The accuracy is always improved, with respect of the non-augmented case, when GEMD and SMOTE are used. This emphasizes the importance of having more data to train the model. Specifically, GEMD shows higher classification accuracy than SMOTE in sessions 2, 3, 5, 6, and 8; while SMOTE has better performance in sessions 1, 4, and 10. In sessions 7 and 9, the accuracies of both GEMD and SMOTE methods are almost the same. In addition, a classification performance of 93.3% is obtained in session 2 by using GEMD, which is the best result obtained with this database so far. The average of the 10 sessions' best accuracy using GEMD achieves 78%, which is better than using SMOTE (74.7%) and the baseline case (55.7%).

### GEMD Performance on Feature Selection Methods

In this section, we evaluate the performance of the GEMD using the procedures described in Zhang et al. (2021). In summary, Zhang et al. proposed an outlier correction on the morphometric features based on the STDC algorithm (Chen et al., 2013) and explored several feature selection methods to classify MRI data from controls and gifted groups. These methods were applied to the UVic-GC dataset with outstanding performance.

According to Zhang et al. (2021), the NONE feature selection method used all the features in the raw feature matrix. The VON feature selection method used only the regions belonging to types 2 and 3 of the von Economo atlas (van den Heuvel et al., 2015), which corresponds to the associative areas of the brain. Choosing the top highest features selected with a threshold, from all the morphometric features and brain regions, was defined as the FS feature selection method. The rank F-score (RFS) method is a variation of the previous one in which, for each region, the FS values are sorted by descending order, where the morphometric features with the highest FS value are the selected ones. Finally, the combination of VON and FS will lead the VFS feature selection method, in which only type 2 and 3 regions are considered when calculating the FS value for morphometric features. Two traditional machine learning methods, KNN and SVM, were used as classifiers (Zhang et al., 2021), with leave-one-out as a cross-validation strategy.

**Figure 8:**
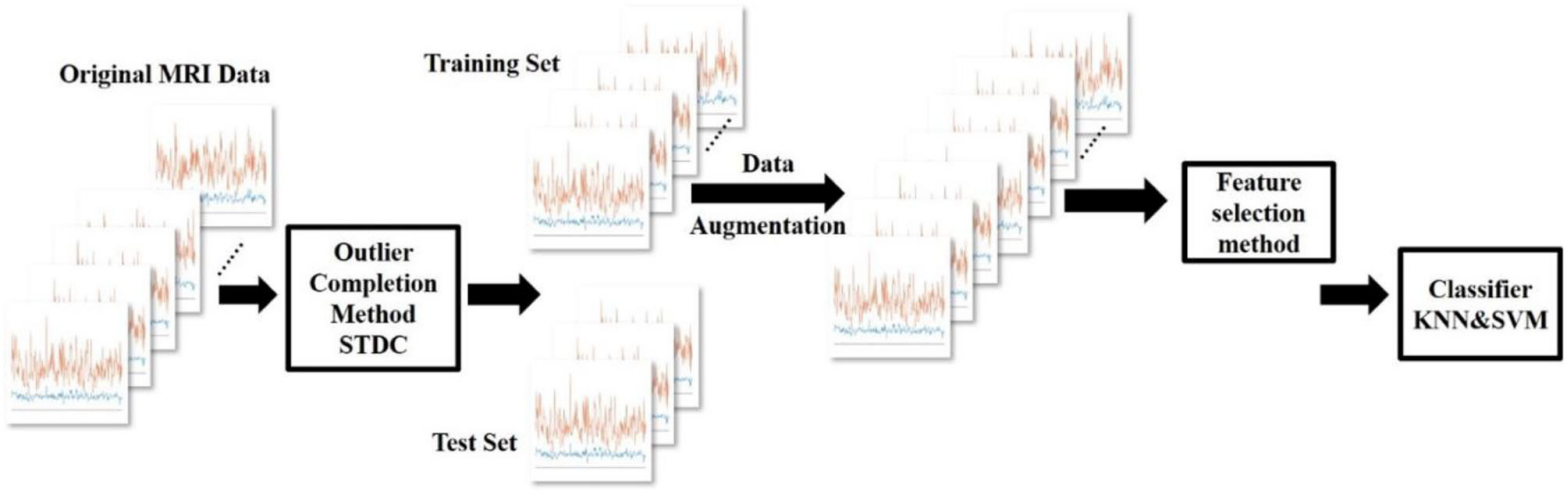
The data augmentation process on the UVic-GC dataset with GEMD and feature selection experiment.

The process of this experiment is shown in Figure 8. First, we use the outlier completion method STDC to compute the missing entries from the estimated latent factors. Then, we enlarge the training set of original MRI data by using GEMD. After that, we use feature selection methods NONE, VON, FS, VFS, and RFS to select different features. Finally, the model is trained by KNN and SVM for classification.

**Figure 9:**
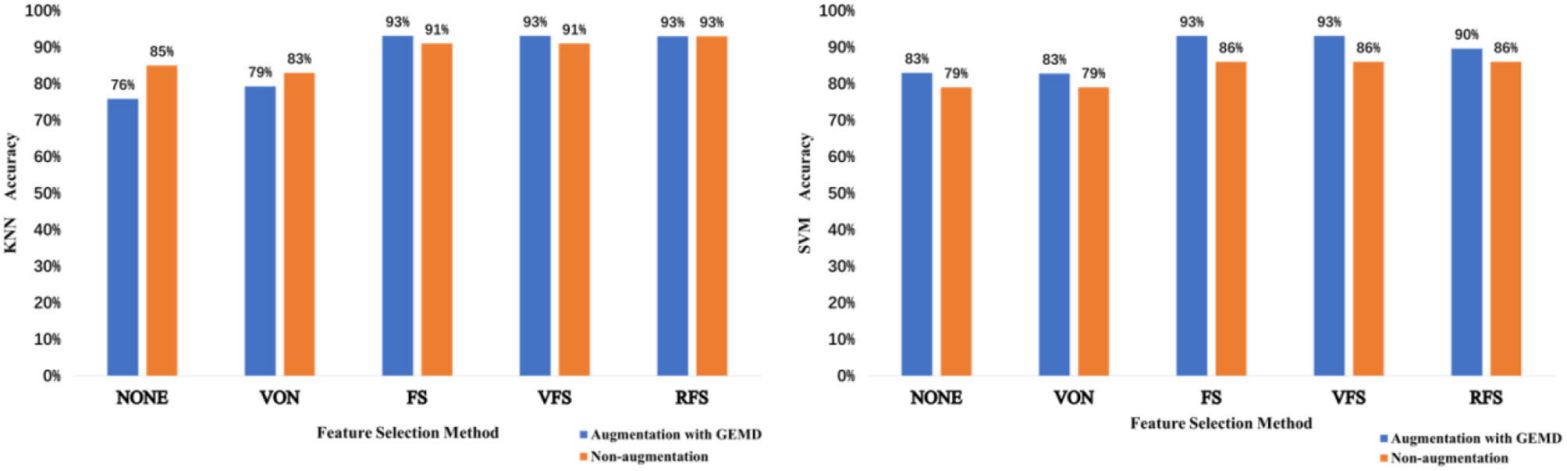
The KNN (left) and SVM (right) accuracies obtained depending on the feature selection method used for the non-augmentation case and augmentation with GEMD case.

From Figure 9, we observe that using data augmentation with GEMD generally improves the performance of feature selection methods. For the SVN case (Figure 9, right), the GEMD method always improves the accuracy regardless of the feature selection method, while for the KNN case (Figure 9, left) only in two cases the accuracy is lower using artificial data. Note that for both KNN and SVM the classification accuracy reaches 93% using FS and VFS, which is the best result with this database, to the best of our knowledge.

## Discussion

In our study, we have used GEMD to enlarge the UVic-GC dataset. The motivation for exploring a data augmentation strategy is 2 fold. First, the UVic-GC dataset is small. Second, there are many parameters in the deep neural network that need to be learned from the data. Therefore, overfitting could appear due to insufficient amount of data.

We propose the GEMD augmentation method to solve the problems mentioned above in this work. We analyze the GEMD augmentation result in three aspects, namely, the influence of the number of artificial subjects, the classification accuracy between non-augmentation and augmentation, and the feature selection method used.

It can be seen from Figure 6 that the accuracy shows an upward trend with the increase in the amount of artificial data. When the number of artificial data reaches 350, the classification accuracy achieves the maximum. Note that the result may vary considerably from experiment to experiment. This is due to the non-convergence of the BrainNetCNN and the random factor added when selecting the data for each experiment. Prettier but unfair results could be shown by discarding the non-convergent experiments, for example, but we show the full set of results to point out these potential problems.

**Figure 10:**
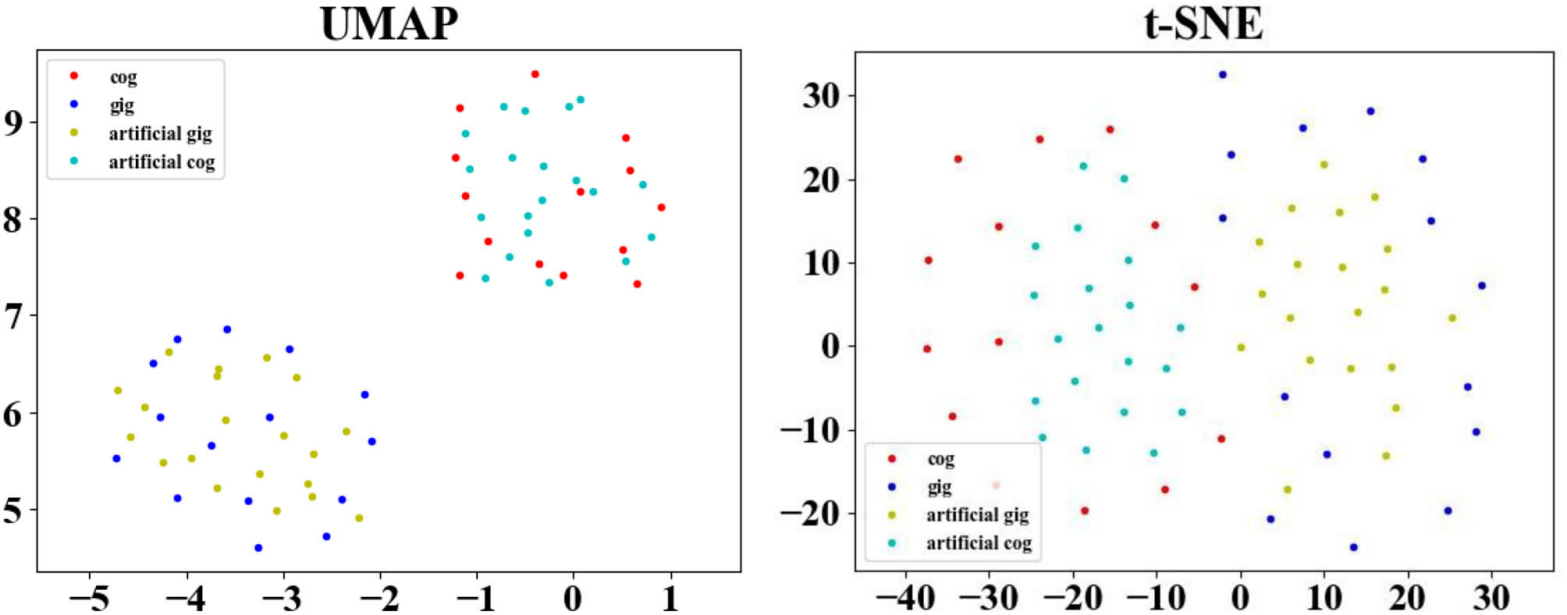
Feature map of the artificial gifted group (artificial gig), artificial control group (artificial cog), original gifted group (gig), and original control group (cog) plotted with UMAP.

To clearly illustrate the distribution of the artificial data generated by GEMD, Figure 10 depicts the original SC matrices, named the original gifted group (gig) and original control group (cog), and 20 artificial SC matrices of the artificial gifted group (artificial gig) and artificial control group (artificial cog). This figure uses Uniform Manifold Approximation and Projection (UMAP) (McInnes et al., 2018) and Distributed Stochastic Neighborhood Embedding (van der Maaten and Hinton, 2008) for dimensionality reduction. It can be seen that the artificial data of each group are projected around the original data of the corresponding group, which is a way of showing that the artificial data are meaningful, i.e., the data generated by GEMD are consistent with the distribution of the original data. Furthermore, the two classes (control and gifted) in the two figures can be accurately separated. There is no obvious overlap between the two groups, explaining why the linear classifiers (SVM and KNN) combined with feature selection methods perform very well.

Even if our proposed method can augment the dataset so that the artificial data help improve the classification accuracy, we must highlight that the results of the BrainNetCNN are not stable. This is due to two main factors, the non-convergence of the model and the overfitting that appears despite the amount of artificial data generated. This is the main drawback of the proposed method. We are now investigating it and other possible neural network models with fewer parameters to improve the classification results when using a small number of original MRI subjects in the training dataset and artificial data generated with them. Figure 10 shows that the artificial data created using the GEMD method are consistent with the original (real) data, which encourages us to use this method and improve the classification model.

## Conclusions

Medical data such as MRI are difficult to obtain, and gifted children are rare in our society. Identifying gifted children from a small set of MRI data is not easy. At the same time, deep neural networks require a large amount of data to improve their performance. They cannot exert their full performance when the dataset is too small. In that case, our work provides a feasible solution by data augmentation. We use the UVic-GC dataset and artificial data generated by GEMD to train the BrainNetCNN neural network. This avoids using a feature selection method as we feed the model directly with the SC data. The results show that GEMD has a significant effect that improves the performance of the classifier. Furthermore, the GEMD data augmentation method can be extended to other similar small datasets. Our future work will focus on the application of GEMD on multisite MRI data, such as the Human Connectome Project data. Due to different scanner settings, parameters, and operators, the distribution of MRI data collected in various regions is different. We expect to be able to adjust the distribution of other datasets by domain adaptation. In that case, we can predict the classification results of multiple MRI datasets using the trained model after augmentation with GEMD.

## Data Availability Statement

The raw anonymized MRI data is available in the OpenNeuro repository: https://openneuro.org/datasets/ds001988. The code for replication is available at: https://github.com/CynthiaChern/GEMD-Based-Data-Augmentation-for-Gifted-Children-MRI-Dataset-Analysis GraphEMD code is available at https://github.com/fkalaganis/graph_emd.

## Ethics Statement

The studies involving human participants were reviewed and approved by Institutional Review Board (IRB00003099) of the University of Barcelona (Catalonia). Written informed consent to participate in this study was provided by the participants' legal guardian/next of kin.

## Author Contributions

ZS, CC, and JS-C: conceptualization. FD, ZS, CC, and JS-C: methodology and supervision. XC, BL, HJ, and FF: formal analysis and investigation. XC: writing. XC, BL, HJ, FF, FD, ZS, CC, and JS-C: writing—review and editing. FD, CC, and JS-C: funding acquisition. FD and JS-C: resources. All authors contributed to the article and approved the submitted version.

## Funding

This study was supported in part by the National Natural Science Foundation of China (Key Program) under Grant No. 11932013 and in part by the Tianjin Natural Science Foundation for Distinguished Young Scholars under Grant No. 18JCJQJC46100. JS-C's work was partially based upon work from COST Action CA18106, supported by COST (European Cooperation in Science and Technology) and the University of Vic – Central University of Catalonia (R0947). CC's work was partially supported by grants PICT 2017-3208, PICT 2020-SERIEA-00457, UBACYT 20020190200305BA, and UBACYT 20020170100192BA (Argentina).

## Conflict of Interest

The authors declare that the research was conducted in the absence of any commercial or financial relationships that could be construed as a potential conflict of interest.

## Publisher's Note

All claims expressed in this article are solely those of the authors and do not necessarily represent those of their affiliated organizations, or those of the publisher, the editors and the reviewers. Any product that may be evaluated in this article, or claim that may be made by its manufacturer, is not guaranteed or endorsed by the publisher.
